# Goal-Directed Therapy: New Trends and Horizons

**DOI:** 10.1155/2012/576253

**Published:** 2012-10-22

**Authors:** Mikhail Kirov, Lars Bjertnaes, Zsolt Molnar, Samir Sakka

**Affiliations:** ^1^Department of Anesthesiology and Intensive Care Medicine, Northern State Medical University, Troitsky Avenue 51, Arkhangelsk 163000, Russia; ^2^Department of Anesthesiology, University Hospital of North Norway, Tromsø, Norway; ^3^Anesthesia and Critical Care Research Group, Department of Clinical Medicine, Faculty of Health Sciences, University of Tromsø, 9037 Tromsø, Norway; ^4^Department of Anaesthesiology and Intensive Therapy, Faculty of Medicine, University of Szeged, Szeged, Hungary; ^5^Department of Anesthesiology and Intensive Therapy, Cologne-Merheim Medical Center, University of Witten/Herdecke, Krankenhaus Merheim Ostmerheimer Str. 200, 51109 Cologne, Germany

In traumatized or critically ill patients suffering from a variety of severe illnesses, survival often depends on the successful handling of a chain of time-critical events with the ultimate goal to provide sufficient tissue oxygenation. Timely initiated goal-directed therapy (GDT) is a cornerstone in the care and treatment of these patients. Based on adequate monitoring techniques, goal-directed algorithms can facilitate the early detection of pathophysiological changes and influence therapies that may improve the clinical outcomes [1]. However, there are still many controversies regarding the application of GDT in different categories of patients [2]. Thus, a search for critical illnesses and conditions that might potentially profit from GDT may be of significant importance. 

In this special issue we have invited authors to submit original research and review articles elucidating the front of research and defining the optimal targets for the management and treatment of various perioperative and critically ill conditions, subsequently describing goal-oriented therapeutical interventions. The papers represent a wide spectrum of topics including early hemodynamic therapy in sepsis, perioperative monitoring, goal-directed hemodynamic optimization in cardiothoracic surgery, goal-oriented management of mechanical ventilation in respiratory failure, and important aspects of novel therapies in acute lung injury and acute respiratory distress syndrome. The publications reflect new therapeutic approaches for many critically ill patients. New targets for GDT and treatment algorithms are elucidated, such as static and dynamic preload parameters, weaning and derecruitment tests during ventilation, esophageal pressure, and other respiratory variables. The papers also reflect the limitations of current monitoring tools and therapeutic strategies and discuss the optimal indications of their use. 

We hope that the articles in this special issue will create new ideas of goal-directed therapies that can be evaluated for future clinical implementation. 

*Mikhail Kirov* *Lars Bjertnaes* *Zsolt Molnar* *Samir Sakka*
